# Unexpected Origins: A Case Report of Escherichia coli-Linked Cerebral Abscess in an Adult

**DOI:** 10.7759/cureus.68592

**Published:** 2024-09-03

**Authors:** Ritika Agarwal, Sharath B Raju, Anand Kalegowda

**Affiliations:** 1 Radiology, M. S. Ramaiah Medical College, Bengaluru, IND

**Keywords:** e. coli: escherichia coli, antimicrobial resistance infectious diseases, ventricular septal defect (vsd), adult congenital heart disease (achd), cerebral abscess

## Abstract

Brain abscesses in patients with congenital heart disease (CHD), particularly cyanotic CHD, present significant morbidity and mortality risks. While *Escherichia coli* is a known neonatal meningitis pathogen, its involvement in adult intracranial abscesses is rare. This report details a 36-year-old female with an uncorrected ventricular septal defect (VSD) and Eisenmenger syndrome, presenting with neurological symptoms. Imaging identified a left cerebral hemisphere ring-enhancing lesion indicative of a brain abscess, and stereotactic aspiration confirmed *E. coli* as the pathogen. Treatment with antibiotics resulted in substantial clinical improvement. This case highlights the rarity of *E. coli* brain abscesses in adults and emphasizes the necessity for early diagnosis and precise microbiological identification to guide effective treatment strategies.

## Introduction

Brain abscesses pose significant morbidity and mortality risks, especially in congenital heart disease (CHD) patients [1]. Incidence varies globally, with 8% in developing countries and 1%-2% in Western countries, and 20% of cases occur without identifiable underlying illnesses or infection sources [2]. CHD represents the most prevalent predisposing factor, with 12.8%-69.4% of pediatric patients with complex CHD developing brain abscesses [3,4] and 25%-46% of unrepaired cyanotic CHD patients experiencing similar complications [5,6].

Despite *Escherichia coli *being a prominent cause of neonatal meningitis in the United States, instances of *E. coli*-associated meningitis beyond infancy are rare, typically occurring post neurosurgery, in trauma, or in cases of hepatic cirrhosis [7]. Intracranial abscesses due to *E. coli* in adults are exceedingly uncommon. This case study highlights the rarity of such infections and underscores the need for the early identification and appropriate treatment of unusual etiologies in intracerebral abscesses, aiming to enhance healthcare professionals' understanding of this condition.

## Case presentation

A 36-year-old female with uncorrected ventricular septal defect (VSD), Eisenmenger syndrome, and seizure disorder presented with fever, vomiting, left-sided headache, right-sided weakness, and episodes of dizziness over five days. The recent discontinuation of antiepileptic medication raised the possibility of seizure-related symptoms. She had a temperature of 100°F, pulse rate of 100 beats per minute (bpm), blood pressure of 126/90 mm Hg, respiratory rate of 24 breaths/minute, and oxygen saturation (SpO_2_) varying between 80% and 83% in room air. Clinical examination showed cyanosis, clubbing, elevated jugular venous pressure, loud P2, an ejection systolic murmur, normal breath sounds, and unremarkable abdominal findings. A neurological examination revealed right-sided hypotonia and decreased power, indicating a focal neurological deficit. Laboratory findings revealed polycythemia with a hemoglobin level of 17.6 g/dL and a hematocrit of 51.8%, along with leukocytosis (total leukocyte count of 13,890 cells/µL and 78.7% neutrophils). Other serum biochemistries were within normal limits. The blood culture did not show any microbial growth. Chest X-ray showed cardiomegaly with increased pulmonary vascular congestion. Echocardiography identified an 18 mm mid-to-basal muscular ventricular septal defect (VSD) with a predominant right-to-left shunt. The pulmonary artery systolic pressure was measured at 105 mm Hg, with a mean pressure of 88 mm Hg (calculated based on peak tricuspid regurgitation velocity and pulmonary regurgitation {PR} gradient). Mild pulmonary regurgitation was noted, along with the significant dilation of the right atrium and right ventricle. Biventricular function was good, with an ejection fraction of 65%. No clots, vegetation, or pericardial effusion was detected during the scan.

**Figure 1:**
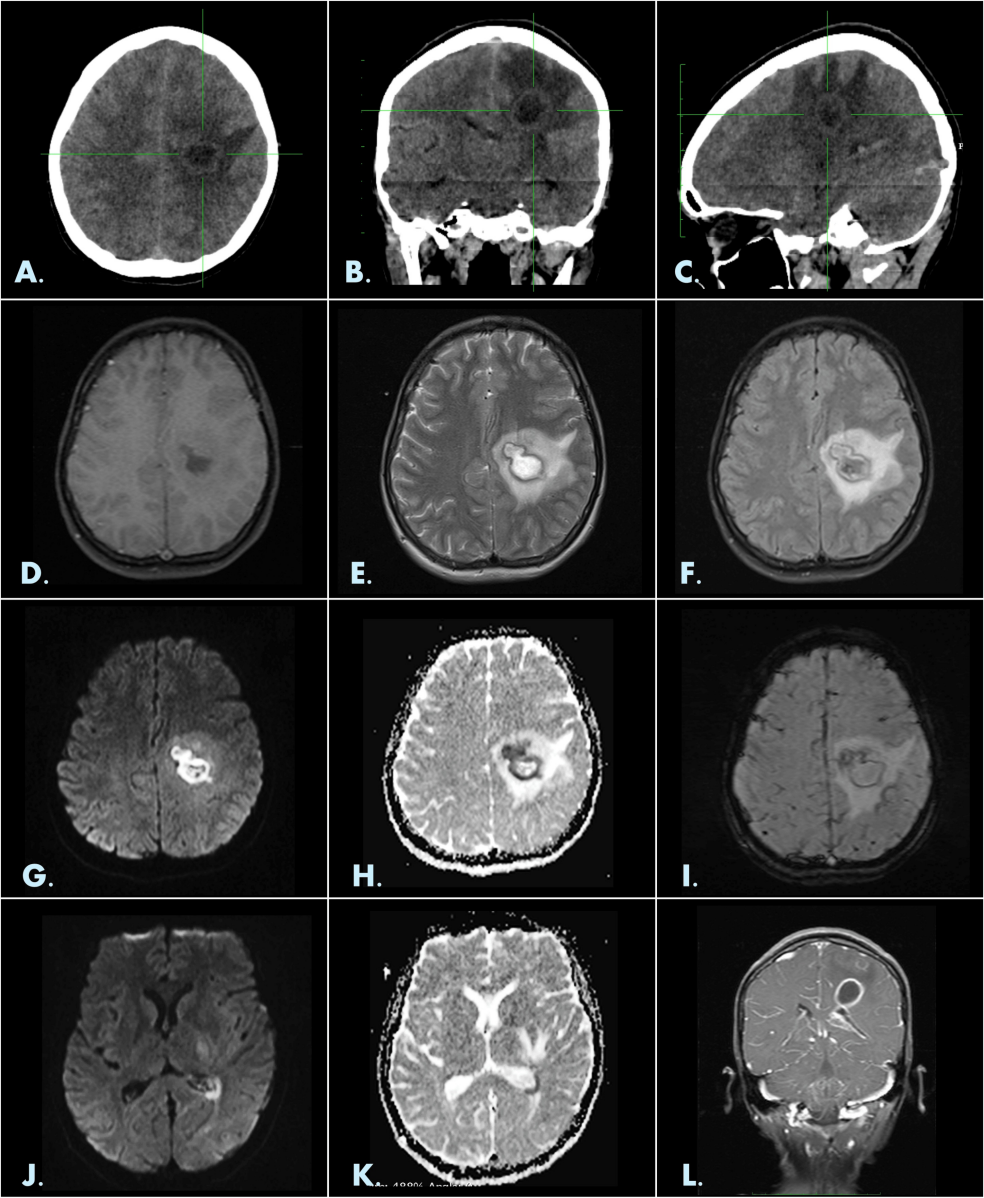
Imaging characteristics of the lesion in the left cerebral hemisphere: CT and MRI findings. Initial non-enhanced CT of the brain showing a 22 × 16 × 18 mm thin-walled lesion with central hypodensity in the left centrum semiovale and corona radiata, accompanied by perilesional edema. Axial (A), coronal (B), and sagittal (C) sections are displayed. MRI images reveal the lesion as hypointense on T1-weighted images (D), hyperintense on T2-weighted images (E), and heterogeneously hyperintense on FLAIR sequences (F). Diffusion restriction is shown (G and H). A dual rim sign is observed on susceptibility-weighted images (I). Additionally, diffusion restriction within the left lateral ventricle suggests rupture into the ventricle (J and K). Post-contrast MRI showed smooth, thin ring enhancement of the lesion with mild enhancement along the ependymal lining of the left lateral ventricle (L). CT, computed tomography; MRI, magnetic resonance imaging; FLAIR, fluid-attenuated inversion recovery

With the suspicion of an acute neurological event in the setting of congenital heart disease, concern for intracranial pathology such as brain abscess or hemorrhage was raised, compounded by the patient's untreated seizure disorder; hence, a computed tomography (CT) of the brain was advised, particularly since blood cultures and echocardiogram were negative for a nidus of infection. CT scan revealed a thin irregular-walled, well-defined lesion with central hypodensity and perilesional edema measuring 22 × 18 × 16 mm in the left centrum semiovale and corona radiata, indicative of a space-occupying lesion. Subsequent contrast-enhanced magnetic resonance imaging (MRI) provided further characterization. The lesion appeared hypointense on T1-weighted images, hyperintense on T2-weighted images, heterogeneously hyperintense on fluid-attenuated inversion recovery (FLAIR) and showed diffusion restriction with perilesional edema causing sulcal effacement and lateral ventricle compression. A dual rim sign was observed on susceptibility-weighted images, characterized by an outer low-intensity rim and an internal high-signal layer. Post-contrast imaging showed smooth, thin ring enhancement of the lesion with mild enhancement along the ependymal lining of the left lateral ventricle. Diffusion restriction within the left lateral ventricle further suggested rupture into the ventricle. These findings were consistent with a left cerebral abscess with ventriculitis (Figure 1).

**Figure 2:**
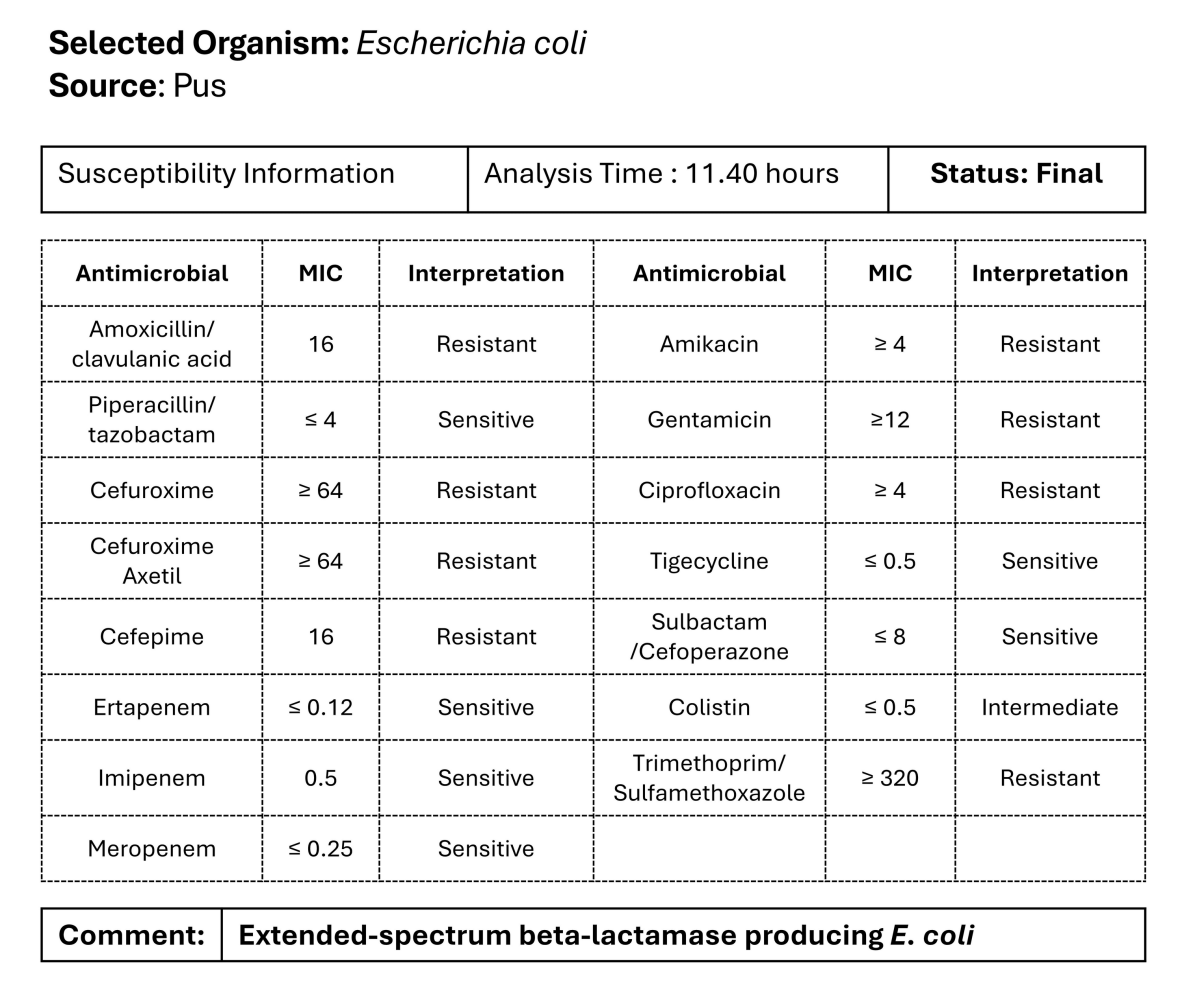
Antibiogram profile of the isolated organism. MIC: minimum inhibitory concentration

The neurosurgery team opted for stereotactic aspiration due to the lesion's size, location, and associated perilesional edema. This minimally invasive procedure aimed to reduce intracranial pressure, obtain a microbiological sample, and enable targeted antibiotic therapy while minimizing damage to surrounding brain tissue. Six milliliters of abscess fluid were aspirated and sent for culture and sensitivity, Gram stain, potassium hydroxide (KOH) mount, acid-fast bacilli (AFB) staining, and anaerobic culture. Gram stain revealed pus cells and necrotic material and a few Gram-negative bacilli. KOH stain was negative for fungal elements, and anaerobic culture showed medium growth of extended-spectrum beta-lactamase (ESBL)-producing *E. coli*. AFB staining was negative. Antibiotic sensitivity testing (Figure 2) guided the initiation of meropenem 1 g every eight hours for three weeks and oral levetiracetam 500 mg twice daily.

**Figure 3:**
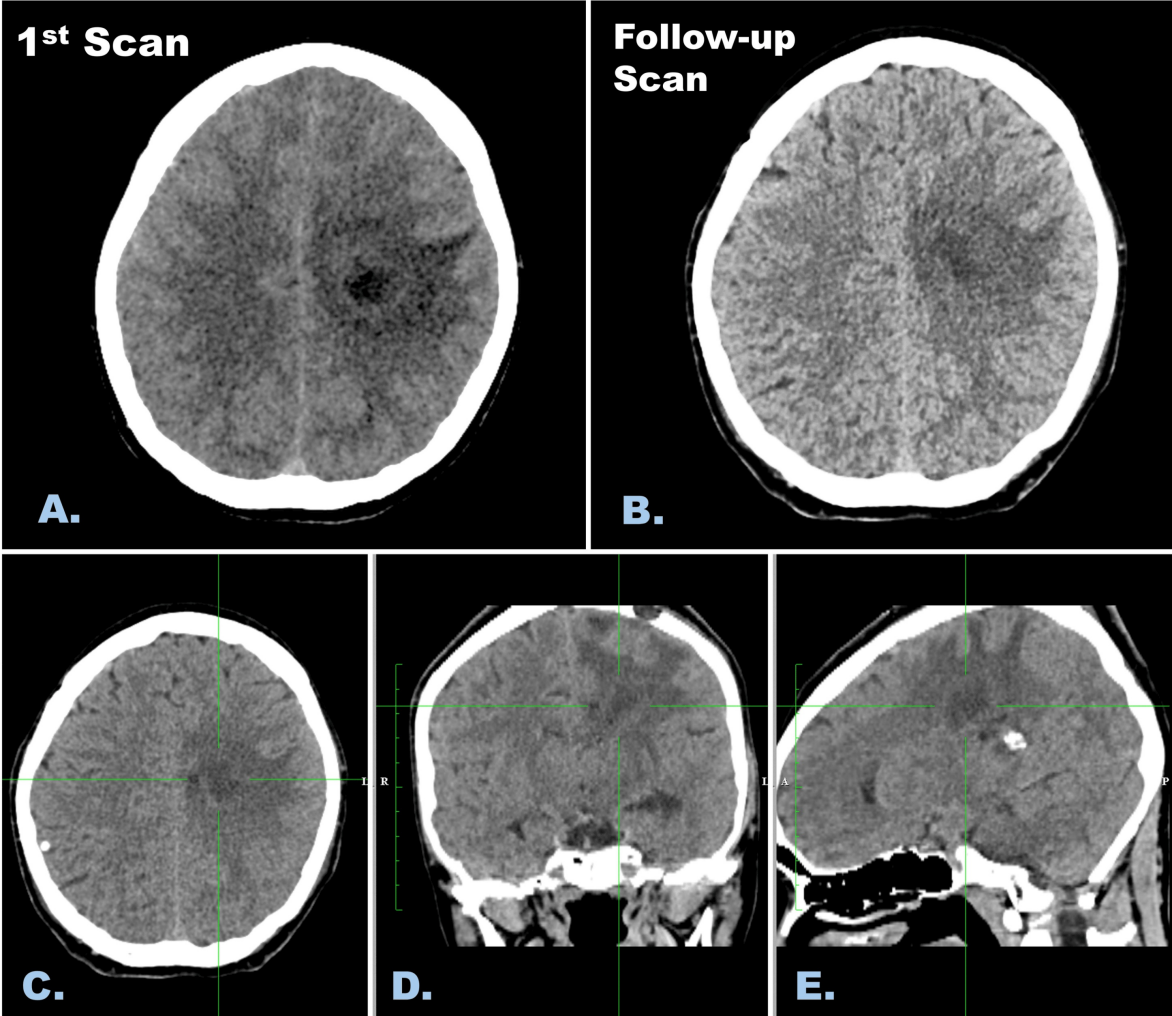
Initial non-enhanced CT of the brain showing lesion in the left centrum semiovale and corona radiata, accompanied by perilesional edema (A). A follow-up non-enhanced CT of the brain after one week showing a significant reduction in the lesion size and perilesional edema (B). Axial (C), coronal (D), and sagittal (E) multiplanar reconstructed images of the follow-up scan. CT: computed tomography

The patient showed significant symptomatic improvement within two weeks. A follow-up non-contrast CT scan of the brain after one week indicated a significant reduction in abscess size and perilesional edema (Figure 3). She was discharged on antibiotics and other precautionary medications. At a two-month follow-up, she was fully recovered with no neurological deficits.

## Discussion

Brain abscesses in patients with cyanotic CHD are multifactorial. The bypassing of pulmonary circulation eliminates alveolar phagocyte filtration, allowing pathogens to enter the systemic circulation and the brain. Severe hypoxemia and metabolic acidosis from secondary polycythemia cause cerebral hypoperfusion, facilitating microbial seeding in under-perfused brain regions. Vascular compromise further predisposes to microthrombus formation and focal infarcts, providing niduses for microbial infiltration and resulting in brain abscess formation [8-10].

In patients with cyanotic CHD, cerebral abscesses are commonly caused by non-hemolytic streptococci, with *Streptococcus milleri* frequently identified [9]. However, in this case, *E. coli* was the causative agent, a rare finding with only two reported cases in the past two decades [11]. Sangi et al. reported a 9.3% incidence of brain abscesses among 544 pediatric patients, predominantly linked to polycythemia, particularly in those over 10 years old. Tetralogy of Fallot was the most common congenital heart defect, with 84.3% of patients having a single abscess and 9.7% of cases caused by *E. coli* [12]. These findings, however, of the pediatric population align with our case, emphasizing the need for targeted management strategies for brain abscesses in CHD patients.

The delayed diagnosis and management of brain abscesses in CHD patients can lead to significant complications, with mortality rates ranging from 27.5% to 71% [10,13]. Larger or deep-seated abscesses necessitate immediate and repeated stereotactic aspiration to alleviate intracranial pressure and obtain microbiological samples for targeted antibiotic therapy based on culture results [14].

Empirical medical therapy is considered adequate for abscesses smaller than 2 cm in diameter, provided the patient is neurologically stable and monitored with serial CT scans [10,15]. Our patient underwent stereotactic aspiration due to the abscess's size and associated ventriculitis. Alternative interventions such as craniotomy and excision are generally avoided due to their poor outcomes, including a mortality rate as high as 71%, and are reserved for cases where conventional approaches fail to control progressive abscesses effectively [15].

This case highlights the critical advantages of multidisciplinary management in optimizing outcomes. An integrated approach involving neurology, cardiology, radiology, microbiology, and neurosurgery is essential to overcome challenges presented by CHD patients with brain abscesses. The early identification of *E. coli* through microbiological examination, which is not a typical pathogen in adult brain abscesses, illustrates the importance of moving beyond purely empirical management strategies. Empirical therapy, while useful in stable cases, may not cover atypical organisms such as *E. coli*, particularly in immunocompromised patients or those with unusual clinical presentations. Microbiological diagnostics facilitated the selection of pathogen-specific antibiotics, optimizing therapeutic efficacy and reducing the risk of adverse effects associated with the indiscriminate use of broad-spectrum antimicrobials. Such an approach ensures timely, targeted, and effective treatment, improving prognosis in this vulnerable patient population.

## Conclusions

Brain abscesses in congenital heart disease, particularly cyanotic forms such as Eisenmenger syndrome, pose significant clinical challenges. This case underscores the crucial role of early diagnosis and management in preventing severe complications and mortality. The identification of *E. coli* as a rare pathogen causing brain abscess emphasizes the importance of considering diverse microbial causes, especially in unusual clinical presentations. Stereotactic aspiration was effective in managing our patient's larger abscess with ventriculitis. This study underscores the necessity for customized therapeutic approaches and careful monitoring to improve outcomes in this vulnerable patient group.
